# Case Report: From somatic pain to shared narrative—mentalization and intergenerational trauma in a transcultural group consultation

**DOI:** 10.3389/fpsyg.2026.1933126

**Published:** 2026-09-11

**Authors:** Iolanda Faustino, Alice Titia Rizzi, Marie Rose Moro, Rahmeth Radjack

**Affiliations:** 1 Hospital D. Estefânia, ULS S. José, Lisbon, Portugal; 2 Maison de Solenn/Maison des Adolescents, Hôpital Cochin, AP-HP, Paris, France; 3 Laboratoire de Psychologie Clinique, Psychopathologie, and Psychanalyse (PCPP), Université Paris Cité, Paris, France; 4 CESP, Inserm U1018, Université Paris-Saclay/UVSQ, Villejuif, France

**Keywords:** adolescent, case report, intergenerational trauma, mentalization, migration, somatic symptoms, transcultural psychiatry

## Abstract

**Introduction:**

Somatic symptoms in migrant and trauma-affected young people often resist a strictly biomedical reading. Trauma, forced migration and family silences may disrupt the parental reflective function, leaving experience unsymbolized and the body as the primary site of expression. Group-based transcultural consultation (TC) has been proposed as a setting able to temporarily sustain mentalization.

**Methods:**

We report the case of Gloria, a young Kinyarwanda-speaking Congolese woman (Banyamulenge) followed since late adolescence after hospitalization for chronic disabling pain with somatoform features alongside ongoing investigation of possible autoimmune findings. Seven TC sessions were conducted between December 2024 and November 2025 at the Maison de Solenn (Cochin Hospital, Paris). The integral verbatim of each session and of the post-session team debriefings was analyzed by the first author under senior-author supervision, using the transcultural analytical framework developed by Maherzi et al. (2024) to identify therapeutic levers. The presentation follows the CARE guidelines.

**Results:**

Gloria’s clinical picture had been shaped by pre-existing somatic and school-related difficulties since childhood, subsequently compounded by a traumatic rupture at age 13—abrupt separation from her mother and three siblings during the South-Kivu conflict—followed by forced migration and 6 years of family separation before reunification in 2023. Three therapeutic processes became visible across the sessions: a movement from a frozen body-bound symptom toward a shared narrative; a movement from mother-daughter affective indifferentiation toward incipient symbolic separation, mediated by the cultural image of the *pagne* (a wax-print cloth of West and Central African tradition carrying meanings of feminine identity and maternal transmission); and the emergence of culturally meaningful representations (Addolorata, Ubuntu, the “pages paires et impaires”—the even and odd pages of a shared family history).

**Discussion:**

The group *dispositif* may be read as a temporary mentalizing envelope in which intergenerational suffering can be progressively named, differentiated and inscribed in a shared cultural and family narrative.

## 1 Introduction

Adolescents and young adults whose suffering is shaped by migration, war exposure and family separation are increasingly encountered in European clinical settings, often expressing distress through bodily symptoms difficult to interpret biomedically (Hinton and Lewis-Fernández, 2011; Ludot-Grégoire et al., 2023). Mentalization theory (Fonagy et al., 2002) describes the capacity to understand one’s and others’ actions as grounded in underlying mental states, developing within a parental reflective function rooted in epistemic trust-the willingness to be open to socially transmitted knowledge from a source perceived as authentic and reliable (Fonagy and Allison, 2014; Campbell et al., 2024). Intergenerational trauma may overwhelm this function: affects circulate undifferentiated, the body carries what cannot be represented—a register that recent formulations approach through embodied mentalizing (Campbell et al., 2024)—and dreaming may collapse (Asen and Fonagy, 2012). In migration contexts, this fragility is compounded by the loss of the cultural world itself (Nathan, 1994; Radjack et al., 2022), and group-based transcultural *dispositifs* may temporarily sustain the reflective function on behalf of an overwhelmed dyad (Moro, 2020).

We report the case of Gloria, a young Kinyarwanda-speaking Congolese woman of the Banyamulenge community, in whom chronic diffuse pain, a traumatic rupture in early adolescence, 6 years of family separation and a complex reunification converged around an explicit therapeutic question about intergenerational transmission. Through a chronological analysis of seven transcultural consultations, we examine how the group setting may have functioned as a mentalizing envelope supporting the transformation of somatic pain into shared narrative.

## 2 Methods

The transcultural consultation (TC) developed by Moro and colleagues is a second-line intervention indicated when standard psychiatric or psychological follow-up reaches its limits-typically when the problem stagnates, when cultural misunderstandings emerge, when the family draws on cultural theories to account for the symptoms, or when migratory trajectories require additional resources (Moro, 2020). It is structured as a group *dispositif*: a principal therapist conducts the encounter and co-therapists of diverse cultural, linguistic and professional backgrounds participate in the propositions and debriefings, an arrangement that encourages clinical decentering. All seven sessions were conducted with a Kinyarwanda-speaking interpreter. Beyond linguistic translation, the interpreter acted as a de facto co-therapist—a full participant in the group’s psychic apparatus (Kaës, 2010) and in the transcultural mediation between systems of meaning (Bouznah and Lewertowski, 2013; Bouznah and Minassian, 2024; Tribe, 2021).

During each consultation, one of the co-therapists transcribes the session and the team’s post-session exchanges word-for-word. This integral verbatim constitutes the material of the analysis. Each session was analyzed by the first author, under senior-author supervision, drawing on the analytical framework developed by Maherzi et al. (2024) to identify therapeutic levers. The framework organizes the reading around three thematic axes: (i) when transmission can be addressed within the encounter-moments propitious to explore family and migratory history; (ii) how therapists encourage transmission-interventions on culture, language and family histories; and (iii) what elements favor its narrativization-conditions supporting the shared construction of the family story. The recurrence and transformation of these therapeutic levers across sessions were then examined longitudinally, allowing us to identify three broader clinical processes that organized the evolution of the case: the shift from body-bound symptom to shared narrative, the movement from mother–daughter indifferentiation toward incipient differentiation, and the emergence of a group dreaming function.

French quotations are attributed to their author; Kinyarwanda was rendered into French through consecutive translation by the interpreter, and every French quotation is followed by an English translation in square brackets. The presentation follows the CARE guidelines (Gagnier et al., 2013).

The patient and her parents gave written informed consent for the publication of anonymized material. The case is reported within the EDPT-ADOS research protocol, approved by the Research Ethics Boards of Sud Mediterranean IV.

## 3 Case presentation

Gloria was first met by the referring child and adolescent psychiatrist (CAP) at 17, during a hospitalization in internal medicine for diffuse disabling pain, at times so intense that she could not get up from bed. The medical assessment suggested a somatoform dimension. Biological anomalies raised the possibility of an autoimmune contribution, but the physical examination was unremarkable, with no focal neurological signs, and the autoimmune workup-still under active investigation at the time of writing-had not confirmed a primary organic etiology. Individual psychiatric follow-up was initiated. The clinical formulation corresponded to a Somatic Symptom Disorder with predominant pain (DSM-5)/Bodily Distress Disorder (ICD-11), with comorbid anxiety features, and progressively oriented toward a traumatic hypothesis. The TC was introduced in December 2024, when Gloria was 20, responding to three converging indications: persistent disabling pain; a working hypothesis of intergenerational trauma reactivated by the family’s recent reunification; and the need to prepare the closing of care by transferring investment from a single therapist toward a group.

Gloria is the second of five siblings of a Banyamulenge family-a Kinyarwanda-speaking population of South Kivu (Democratic Republic of the Congo), historically marginalized and exposed to cycles of persecution, particularly after the 1994 Rwandan genocide. Since childhood she had presented with visual difficulties, fatigue, memory problems, recurrent vertigo and occasional loss of consciousness.

In 2017, when Gloria was 13, the family’s village was attacked. Gloria, her father and her older sister crossed Lake Tanganyika, traveled through Tanzania and reached Mayotte before arriving in France; her mother and the three younger siblings escaped through the forest into Uganda and were received in Nyakabande refugee camp for 2 to 3 years. The family was reunited in France in 2023, 6 years after separation, and identifies with Evangelical Christianity.

At referral to TC, Gloria presented with chronic diffuse pain, severe school anxiety and significant functional impairment. The team identified a notion of possible anxious school refusal pre-dating the 2017 migration. Tables 1 and 2 summarize the seven consultations and the episode-of-care timeline.

**Table 1:** Overview of the seven transcultural consultations (family attendance, concurrent care, main theme, and one illustrative verbatim per session with English translation).

Session	Family present	Interpretation	Concurrent therapies	Main clinical theme	Illustrative verbatim [English translation]
TC1 (Dec 2024)	Gloria, mother	Kinyarwand-French	Individual psychiatric follow-up	Frozen narrative; pain as shared object	*“je le ressens d’une façon double*” [I feel it doubly]
TC2 (Feb 2025)	Gloria, mother	Idem	Individual psychiatric follow-up	Religious framings; pain and prayer; dream asymmetry	*“j’ai fui la guerre mais ici je la continue*” [I fled the war but here I continue it]
TC3 (Apr 2025)	Gloria, mother	Idem	Individual psychiatric follow-up; *soins-études* day hospital for chronic pain	Group begins to dream on Gloria’s behalf: crazy race; Addolorata; ritual of separation	Group’s dreaming function (no single verbatim)
TC4 (Jun 2025)	Gloria, mother	Idem	Individual psychiatric follow-up; *soins-études* (ongoing)	Interpreter’s dream; Ubuntu; emergence of Gloria’s first dream	*“mon rêve c’est ubuntu… si vous êtes là, rien ne peut arriver*” [my dream is ubuntu… if you are there, nothing can happen]
TC5 (Jul 2025)	Gloria, mother	Idem	Individual psychiatric follow-up; end of *soins-études*; return home	The pagne as maternal transmission; recovery of dreaming; 2021 paralysis story; prayer replaces analgesics	*“Que tout est possible… la nuit aussi*” [That all is possible… at night too]
TC6 (Sep 2025)	Gloria, mother	Idem	Individual psychiatric follow-up	School suffering; the body in transformation (pain, haircut, adolescence); words as medicine; mother’s distinct position	*“le meilleur médicament pour moi c’est la parole*” [the best medicine for me is the spoken word]
TC7 (Nov 2025)	Gloria, father (1st attendance)	Idem	Individual psychiatric follow-up; enrolment in Red Cross program	Paternal narrative; a second given name; even and odd pages; “Grâce à Dieu” [Thanks be to God]	*“Aujourd’hui on lit les pages impaires, et avec sa mère on lisait les pages paires*” [Today we read the odd pages; with her mother we used to read the even pages]

**Table 2:** Episode-of-care timeline (CARE guidelines, item 7): key events in Gloria’s history from childhood to the transfer of care in January 2026.

**Date / period**	**Event**
Childhood (before 2017)	Visual difficulties, fatigue, memory problems, recurrent vertigo, occasional loss of consciousness; possible anxious school refusal; long-standing lack of confidence.
2017 (age 13)	Attack on the family village in South Kivu; separation from mother and three younger siblings; migration with father and older sister across Lake Tanganyika and Tanzania to Mayotte, then France.
2017–2023	Family separation; mother and younger siblings in Nyakabande refugee camp (Uganda); Gloria in France.
2021 (age 17)	Hospitalization in internal medicine for diffuse disabling pain; medical workup identifies somatoform dimension and possible autoimmune contribution; individual psychiatric follow-up initiated. Episode of paralysis resolved after several days of family prayer.
2023	Family reunited in France after 6 years of separation.
Dec 2024 (age 20)	TC1 - first transcultural consultation; frozen narrative.
Feb 2025	TC2 - religious framings; pain and prayer.
Apr 2025	TC3 - group begins to dream on Gloria’s behalf. Admission to *soins-études* day hospital for chronic pain
Jun 2025	TC4 - interpreter’s dream; Ubuntu; Gloria’s first formulated dream.
Jul 2025	TC5 - end of *soins-études*; return home; prayer replaces analgesics; recovery of dreaming; 2021 paralysis story.
Sep 2025	TC6 - school suffering; body in transformation; words as medicine; mother’s distinct position.
Nov 2025	TC7 - father attends for the first time; paternal narrative; a second given name; even and odd pages; enrolment in a Red Cross program.
Jan 2026	Final individual psychiatric follow-up; transfer of care to the Minkowska Centre; gifts brought by the family; Ubuntu photograph to be framed or painted in the country of origin.

## 4 The therapeutic process

### 4.1 First consultation (December 2024): the frozen narrative

The first consultation explored the family composition, the separation, the migratory journey and the onset of pain. Gloria’s narrative appeared frozen; much of the history was carried by her mother, who described her daughter as silent and withdrawn since infancy. The mother then articulated the affective bond explicitly: “La douleur de Gloria me touche avant qu’elle ne sente sa douleur. Ce qu’elle ressent je le ressens d’une façon double” [Gloria’s pain reaches me before she feels her own; what she feels, I feel doubly]. The pain seemed to circulate as a shared object rather than to belong to one of them in particular.

### 4.2 Second consultation (February 2025): religious framings

The family reported an intensification of Gloria’s pain crises after a trip to Rwanda. Gloria relied on prayer; her mother disclosed an active religious dream life and revealed, until then unknown to her daughter, that she spread the Gospel within the community. Gloria reported that she did not dream and was afraid to sleep. This asymmetry opened a clinical space in which what was shared and differentiated between mother and daughter could begin to emerge. The group framed Gloria’s pain as a possible initiatory path within the family’s spiritual lineage, and Gloria engaged with unusual openness: “j’ai fui la guerre mais ici je la continue” [I fled the war but here I continue it].

### 4.3 Third consultation (April 2025): when the group begins to dream

Gloria reported feeling more stable. She had been hospitalized at a *soins-études* day hospital – a therapeutic day-hospital combining medical and psychological care with schooling—for her chronic pain, and described the hospital and consultation group as a “deuxième famille” [second family]. Since Gloria remained unable to access dream activity, the group began to assume a dreaming function on her behalf, offering three representational forms: a “crazy race” framing the pain as the trace of an exhausting migratory journey; the name Addolorata, from a Biblical tradition of names given in periods of collective catastrophe; and a ritual of separation to allow mother and daughter to externalize what each carried.

### 4.4 Fourth consultation (June 2025): Ubuntu and the emergence of a dream

The fourth consultation opened with a dream that the interpreter had had between sessions and shared with the team, in keeping with the *dispositif’s* recognition of clinician counter-transferential material (Lachal, 2006). The team read it as a dream of expulsion, giving form to the image of pain as something foreign to Gloria, transmitted rather than properly hers, that could leave the body. Gloria then asked the team to translate Ubuntu, written on a decorative board in the consultation room alongside African and French cultural elements. The group explained Ubuntu as a philosophy of humanity and interdependence (“Je suis ce que je suis grâce à ce que nous sommes tous” [I am who I am thanks to who we all are]). Until then distracted, Gloria formulated a personal insight:

*“Pendant que vous expliquiez, j’étais distraite et je regardais le tableau. J’ai compris quelque chose sur ubuntu… Mon rêve c’est ubuntu… Quand je vous vois entourée, c’est la grâce de Dieu. Vous cherchez une solution pour moi. Si vous êtes là, rien ne peut arriver”.*

[While you were explaining, I was distracted and looking at the board. I understood something about ubuntu… My dream is ubuntu… When I see you gathered around, it is the grace of God. You are looking for a solution for me. If you are there, nothing can happen.]

For the first time across the sessions, Gloria moved from an absence of dreams to a formulated dream—not of personal achievement but of relational protection. The session ended with a collective photograph under the word Ubuntu.

### 4.5 Fifth consultation (July 2025): dreams, prayer and the weaving of one’s own pagne

Gloria arrived with a new haircut and a more confident posture, having returned home at the end of her *soins-études* program. She still experienced pain episodes but now used prayer in place of analgesics. The principal therapist offered a synthetic formulation: “C’est comme si la douleur était un marqueur de ce que tu ressens” [It is as if the pain were a marker of what you feel]. Asked about her dreams, Gloria replied for the first time “Que tout est possible” [That all is possible], specifying “la nuit aussi” [at night too]. She also narrated an episode of paralysis in 2021 resolved after several days of family prayer, which she read as a vocation to serve God. The group’s propositions drew on the image of the pagne-a wax-print cloth of West and Central African tradition carrying meanings of feminine identity and maternal transmission—suggesting that Gloria was beginning to weave her own.

### 4.6 Sixth consultation (September 2025): school, the body, and words as medicine

Gloria spoke for the first time of school-related suffering: a change of school at age 11 she had never understood, and teachers who had told her she was “good for nothing”—a memory that returned in pain episodes. The mother now adopted a more active maternal stance, describing her parenting as “à la manière militaire” [military fashion], and reminding Gloria that the family supported her and that above her was God. Gloria reported dreaming every night. The group’s propositions circled around three images: pain as the beginning of cicatrization; the haircut as a ritual of renewal; and adolescence as a ritual of passage. Gloria confirmed: “le meilleur médicament pour moi c’est la parole” [the best medicine for me is the spoken word]. The mother situated her own position outside the ancestor frame: “je dialogue avec les personnes physiques que je vois, je ne pense pas aux anciens” [I dialogue with the physical people I see, I do not think of the ancestors].

### 4.7 Seventh consultation (November 2025): the father’s narrative and the missing pages

The seventh consultation was marked by the presence of the father, who had not previously joined the group. Gloria presented with general clinical improvement and had recently joined a Red Cross program toward qualifying as a medical secretary. The father situated the family within the Banyamulenge community of South Kivu and recalled that on the day of the attack he had been with Gloria and her older sister at a church choir gathering. He added that Gloria had already presented health difficulties before the 2017 attack, and that in the years of family separation the family had feared she would not survive. The group’s propositions moved around three figures. Drawing on the French psychoanalytic concept of empathie métaphorisante (Lebovici, 1998), a co-therapist hypothesized that Gloria might bear a second given name—an anticipation the father confirmed, designating Gloria as her father’s daughter. Another co-therapist offered: “Aujourd’hui on lit les pages impaires, et avec sa mère on lisait les pages paires” [Today we read the odd pages, and with her mother we used to read the even pages]; Gloria agreed that the pages had to be brought together. Finally, Gloria’s invocation of “Grâce à Dieu” [Thanks be to God] was re-read as a space able to hold both love and anger; the father echoed this but, pressed further, replied that suffering had never led him to doubt, and Gloria aligned with her father, reaffirming gratitude to God.

## 5 Follow-up and outcomes

Gloria’s chronic pain did not disappear, but by the fifth session she used prayer in place of analgesics and by the seventh pain had a smaller impact on her daily functioning. Dream activity evolved from initial absence and fear of sleep to daily oneiric activity by the sixth and seventh sessions. The mother–daughter relationship moved from indifferentiation toward incipient differentiation, and Gloria moved from silence to authoring her own narrative. By November 2025 she had joined a Red Cross preparatory program. In the final individual follow-up, in January 2026, Gloria and her family brought gifts to the team and asked whether the Ubuntu photograph could be framed or painted in their country of origin; within the family’s cultural register, declining was not clinically tenable. These gestures marked the progressive closing of the care relationship and an emerging reciprocity within it (Zielinski, 2011); care was subsequently transferred to the Minkowska Centre for continuation of individual follow-up.

Across the seven consultations, Gloria progressively took the group’s propositions into her own words – “j’ai fui la guerre mais ici je la continue” [I fled the war but here I continue it] in the second session, “mon rêve c’est ubuntu… si vous êtes là, rien ne peut arriver” [my dream is ubuntu… if you are there, nothing can happen] in the fourth, and “le meilleur médicament pour moi c’est la parole” [the best medicine for me is the spoken word] in the sixth. Adherence was complete and no adverse or unanticipated events were reported by the patient, her family, or the clinical team over the eleven-month period.

## 6 Discussion

### 6.1 From somatic expression to symbolic narration

Tomasella (2013) describes trauma as involving three interdependent dimensions: a loss of continuity of existence, an inability to metabolize lived experience, and the imminent danger of losing one’s human references. When extreme violence and forced displacement disrupt these references, what cannot be metabolized may return through bodily sensations (van der Kolk, 2014). In transcultural situations, the coexistence of biomedical, religious and ancestral explanatory systems may offer pathways through which suffering becomes intelligible. Gloria’s trajectory illustrates one such pathway: pain progressively shifted from isolated bodily intrusion to a signifier of affect that could be put into words. The principal therapist named this shift – “c’est comme si la douleur était un marqueur de ce que tu ressens” [it is as if the pain were a marker of what you feel]—and by the sixth session Gloria took it up as her own: “le meilleur médicament pour moi c’est la parole” [the best medicine for me is the spoken word]. These registers are complementary rather than competing: the medical findings (an autoimmune workup still ongoing), the psychiatric diagnosis (Somatic Symptom Disorder with predominant pain/Bodily Distress Disorder), the culturally shaped idiom of distress (Hinton and Lewis-Fernández, 2011; Kirmayer et al., 2014), and the psychodynamic-transcultural interpretation proposed here. In psychoanalytic terms, the body carried unmetabolized beta-elements (Bion, 1962), awaiting the primary symbolization (Roussillon, 1999) that the group came to support; in transcultural terms, pain functioned as a shared idiom through which the family’s history could be addressed.

### 6.2 The transcultural group as a mentalizing envelope

Mentalization theory (Fonagy et al., 2002; Campbell et al., 2024) holds that the capacity to represent internal states develops within the parental reflective function, rooted in epistemic trust (Fonagy and Allison, 2014). Intergenerational trauma may overwhelm this function: affects remain captured in the parent’s distress, the body carries what cannot be represented, and dreaming may collapse. In Gloria’s case, the mother’s “je le ressens d’une façon double” [I feel it doubly] suggested that pain circulated undifferentiated, while the dream asymmetry pointed to a specific impasse of mentalization on Gloria’s side.

The expression “mentalizing envelope” articulates two complementary traditions: the theory of mentalization and epistemic trust (Fonagy et al., 2002; Fonagy and Allison, 2014), which describes the reflective function that intergenerational trauma may overwhelm; and the psychoanalytic lineage of containment and psychic envelopes, which specifies the intersubjective and group conditions under which such a function can be temporarily lent. Bion’s (1962) model of the container–contained and of reverie names the elementary operation: an alpha function that transforms raw, unrepresented sensory and affective elements—Gloria’s pain and her dreamless nights—into elements that can be dreamt, thought and put into words. What we have called the group’s “dreaming function” is this alpha function performed, for a time, at the group level rather than the individual.

The notion of envelope draws on Anzieu (1985) Moi-peau and the psychic-envelope tradition (Anzieu et al., 1987)—a containing psychic skin holding, inscribing and differentiating an inside from an outside. Clinically, this envelope comprised identifiable elements: the stability of the setting; the plurality of co-therapists with their diverse cultural and linguistic worlds; the continuous presence of the same interpreter; the board bearing African and French cultural references; and the circulation of affects and images among all participants. Within this frame the group functioned as a group psychic apparatus (Kaës, 2010)—where unconscious work is distributed and transference diffracted across several figures—and as a group matrix (Foulkes, 1984), a shared network of communication in which meaning is co-constructed. The interpreter’s own dream, brought to the team between the third and fourth sessions and worked with as counter-transferential material (Lachal, 2006), belonged to the group apparatus rather than to one clinician; through the group’s elaboration, the image of pain foreign to Gloria—transmitted rather than properly hers, and able to leave the body—became available to her.

These processes are distinct rather than equivalent. Following Roussillon (1999), the movement from a body-bound symptom to shared narrative can be read as a passage from primary to secondary symbolization: the group first offered pictographic and sensory supports that gave primary form to what was not yet representable, before these images could be woven into narrative. Rather than successive stages of a single developmental sequence, these concepts are better understood as complementary and partly overlapping lenses on the same clinical material: containment (Bion, 1962), the psychic envelope (Anzieu, 1985), primary symbolization (Roussillon, 1999), narrative elaboration and reflective functioning (Fonagy et al., 2002) each foreground a different facet of how bodily and sensory experience may become representable. We advance their articulation as a clinically grounded hypothesis rather than a linear model: in Gloria’s trajectory they appeared less as a fixed sequence than as intertwined registers of one, non-linear movement toward symbolization within the group.

The transcultural group may have temporarily performed this reflective function on her behalf (Asen and Fonagy, 2012), offering symbolic forms—the “crazy race”, Addolorata, the ritual of separation, Ubuntu, the pagne, and the “pages paires et impaires” [the even and odd pages]—through which the pain could be contained and represented (Lebovici, 1998; Zempléni, 1983). A specific feature of this envelope is its relational ontology, captured in Ubuntu—“Je suis ce que je suis grâce à ce que nous sommes tous” [I am who I am thanks to who we all are]—which coincides with what the transcultural group performs. When Gloria said “mon rêve c’est ubuntu… si vous êtes là, rien ne peut arriver” [my dream is ubuntu… if you are there, nothing can happen], she did not merely adopt a concept: she named the dispositif itself as her own. The emergence of the 2021 paralysis story in the fifth session and the recovery of daily oneiric activity by the sixth and seventh suggest that this borrowed mentalizing work had progressively rendered Gloria’s own history thinkable in the first person (Moro, 2006).

### 6.3 Group processes and the transcultural specificity of the dispositif

The symbolic forms that supported this work—the *pagne*, Ubuntu, Addolorata, Gloria’s second given name, the “even and odd pages”—did not pre-exist the sessions as fixed cultural meanings. Each emerged within the group as what Foulkes (1984) termed a matrix: a shared network of communication in which meaning is co-constructed and no single participant is its sole author. Because the co-therapists, the interpreter and the family each spoke from a different cultural and linguistic position—Kinyarwanda and Banyamulenge, French, the Evangelical register—every representation was proposed, translated, taken up or set aside rather than imposed. When Gloria said “mon rêve c’est ubuntu” [my dream is ubuntu], she was not receiving a cultural attribution but appropriating a proposition that had circulated among several voices. Holding these systems of meaning open at once, without collapsing the family into a single, homogeneous “culture,” defines the transcultural setting (Nathan, 1994; Moro, 2020).

This distinguishes the dispositif from a group psychoanalysis conducted with a migrant family. Rooted in Devereux’s ethnopsychiatry and formalized in France by Nathan (1994) and Moro (2020), the transcultural consultation makes the family’s languages, aetiological theories and cultural representations into therapeutic levers in their own right, and relies on cultural decentering as an active principle (Bouznah and Lewertowski, 2013; Bouznah and Minassian, 2024). In Gloria’s case this cultural material was clinically operative rather than illustrative: the Kinyarwanda mediation, the *pagne* as a support of maternal transmission, the tradition of names given in times of collective catastrophe, the paternal given name that inscribed Gloria within a lineage, Ubuntu as a relational ontology, and the family’s faith set against the history of persecution of the Banyamulenge of South Kivu—each did part of the containing, symbolizing and differentiating work described above. The articulation of group psychic processes with this culturally mediated elaboration—neither reducible to the other—gives the transcultural group its specific therapeutic action.

More precisely, the therapeutic action of the *dispositif* may be understood as emerging from the articulation of two envelopes. The first—a group envelope—is not specific to the transcultural setting: the relational, affective and representational holding offered by the plurality of therapists, the interpreter and the stability of the frame can be found in group analysis more broadly (Foulkes, 1984; Kaës, 2010). The second—a cultural envelope, in the lineage of Anzieu’s (1985) containing envelope—is what the transcultural consultation specifically adds: its contents (the *pagne*, Ubuntu, the second given name, the “even and odd pages”) do not pre-exist as fixed elements to be applied but are translated, negotiated and appropriated through decentering and mediation between the family’s systems of meaning and those of the therapists, the interpreter and the group. It is the articulation of these two envelopes—the general group envelope and the transcultural-specific cultural envelope—rather than either alone, that appears to characterize the specific therapeutic function of transcultural group consultation.

### 6.4 Intergenerational transmission and the work of differentiation

Trauma may be transmitted within the mother–daughter dyad through affect, body and silence (Lebovici and Stoléru, 1983). When such transmission goes unrecognized, the child’s psychic life may organize itself around what cannot be metabolized in the parent—what Feldman et al. (2019) call “radioactive residues”. In Gloria’s case, this was captured in the mother’s “je le ressens d’une façon double” [I feel it doubly] and in the clinical observation that pain circulated as a shared object; the fourth-consultation proposition that pain was foreign, transmitted rather than properly hers, and could leave the body named this register in a form Gloria could take up.

The therapeutic work supported a progressive differentiation. The ritual of separation, the framing of pain as foreign, and the image of the pagne offered symbolic forms through which mother and daughter could externalize what each carried. Beyond the dyad, the inscription of the symptom within a wider family and cultural history—the Banyamulenge lineage and the religious framework voiced by the father in the seventh session—allowed it to be recognized and shared. Maherzi et al. (2024) describe such interventions as conditions for “conscious transmission”—what is carried can be known, named and in part refused, rather than silently reduplicated. By the sixth consultation the mother had asserted her own subjective position (“je dialogue avec les personnes physiques que je vois, je ne pense pas aux anciens” [I dialogue with the physical people I see, I do not think of the ancestors]); by the seventh, the father’s narrative added a third voice, allowing Gloria to be recognized within a lineage in which several differentiated positions could coexist.

### 6.5 Strengths and limitations

The case rests on rich longitudinal material—verbatim transcripts and team debriefings across seven sessions. Limitations should be acknowledged. The reading operates within a specific theoretical orientation (transcultural psychoanalytic, with mentalization as a reading lens); other orientations would foreground different features. The verbatim cited are mediated by consecutive Kinyarwanda-French interpretation, an intrinsic feature of the dispositif. The clinical evolution was shaped by concurrent factors (individual follow-up, medical care, the *soins-études* programme, the family reunion) whose contributions cannot be disentangled. As a single case, no generalizability is claimed; findings are offered as hypotheses to be tested in further single-case and group studies in comparable transcultural settings.

## 7 Conclusion

Gloria’s case illustrates how chronic somatic pain in a young adult with childhood difficulties, war exposure and forced migration may require an integrated medical, psychological, familial and transcultural formulation. Within the containing and dreaming function of the transcultural group, the symptom appeared to shift progressively from bodily intrusion toward shared narrative, from mother-daughter indifferentiation toward incipient symbolic separation, and from absent dreams toward a daily oneiric activity inscribed in a family lineage. Group-based transcultural consultation may offer a useful mentalizing envelope for young people whose trauma remains suspended between body, family and exile. This proposition is offered as a clinically grounded hypothesis, to be confirmed and refined through further single-case and comparative studies in transcultural settings.

## Data Availability

The original contributions presented in the study are included in the article; further inquiries can be directed to the corresponding author.
